# Optimization of hierarchical structure and nanoscale-enabled plasmonic refraction for window electrodes in photovoltaics

**DOI:** 10.1038/ncomms12825

**Published:** 2016-09-26

**Authors:** Bing Han, Qiang Peng, Ruopeng Li, Qikun Rong, Yang Ding, Eser Metin Akinoglu, Xueyuan Wu, Xin Wang, Xubing Lu, Qianming Wang, Guofu Zhou, Jun-Ming Liu, Zhifeng Ren, Michael Giersig, Andrzej Herczynski, Krzysztof Kempa, Jinwei Gao

**Affiliations:** 1 Institute for Advanced Materials and Guangdong Provincial Key Laboratory of Quantum Engineering and Quantum Materials, South China Normal University, Guangzhou 510006, China; 2 Department of Physics, Freie Universität Berlin, 14195 Berlin, Germany; 3 Max Planck Institute of Colloids and Interfaces, 14476 Potsdam, Germany; 4 Department of Physics, Boston College, Chestnut Hill, Massachusetts 02467, USA; 5 Electronic Paper Displays Institute, South China Normal University, Guangzhou 510006, China; 6 School of Chemistry and Environment, South China Normal University, Guangzhou 510006, China; 7 Laboratory of Solid State Microstructures, Nanjing University, Nanjing 210093, China; 8 Department of Physics and TcSUH, University of Houston, Houston, Texas 77204, USA; 9 Helmholtz-Zentrum Berlin, Institut Nanoarchitekturen für die Energieumwandlung, 14109 Berlin, Germany

## Abstract

An ideal network window electrode for photovoltaic applications should provide an optimal surface coverage, a uniform current density into and/or from a substrate, and a minimum of the overall resistance for a given shading ratio. Here we show that metallic networks with quasi-fractal structure provides a near-perfect practical realization of such an ideal electrode. We find that a leaf venation network, which possesses key characteristics of the optimal structure, indeed outperforms other networks. We further show that elements of hierarchal topology, rather than details of the branching geometry, are of primary importance in optimizing the networks, and demonstrate this experimentally on five model artificial hierarchical networks of varied levels of complexity. In addition to these structural effects, networks containing nanowires are shown to acquire transparency exceeding the geometric constraint due to the plasmonic refraction.

## Abstract (web summary)

In photovoltaics window electrodes must display uniform current transport, as well as high light transmission from the substrate. Here, Han *et al.* show that quasi-fractal metallic networks provide a practical realization of an electrode structure with an optimal surface coverage and a uniform current density.

## Graphical abstract

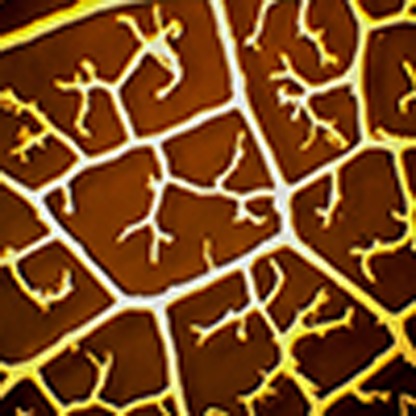

High electrical conductivity and optical transmittance of window electrodes play a crucial role in various optoelectronic devices such as solar cells^1^,^2^,^3^, light-emitting diodes (LEDs)^4^,^5^,^6^, displays^7^,^8^,^9^, sensors^10^,^11^,^12^ and smart windows^13^,^14^,^15^. In photovoltaic (PV) applications (solar cells and LEDs), window electrodes must display an efficient and uniform current transport, as well as a very high light transmission into/from the semiconductor substrate^16^,^17^.

Various window electrode materials have been developed in the last century, which have been based on thin, uniform films of metal oxides, highly doped large gap semiconductors. The most common of those today is the indium tin oxide (ITO). The need for ITO replacement has recently emerged due to relatively high cost, shortage of indium, brittleness and the fact that transparency and the overall conductivity is not very good. In addition, these materials are continuous, uniform films, and thus not optimal for collection of non-uniform currents developing around contacts, and other non-uniformities in the PV structures, thus affecting their efficiency.

In this context, it has been argued that quasi-fractal (QF) or hierarchical metallic networks could be a good ITO replacement, optimal for the required function of efficient and uniform current transport, as well as a very high light transmission into/from the semiconductor substrate^18^. In an analogous problem of the heat flow, it was demonstrated that QF structures lead to a marked reduction of the thermal resistance^19^. A similar problem of nutrient delivery into a leaf's tissue, with minimal light shading, has been solved by the natural evolution with a QF hierarchical network of the leaf-venation system^18^,^19^,^20^,^21^. It is also known that river tributaries from a basin develop a self-similar structure of branching to homogenize water flow^22^.

In this work, we first propose a theoretical analysis of window electrode networks subject to the following optimization conditions: (i) a maximal surface coverage; (ii) a uniform current density; and (iii) a minimum of the overall resistance at fixed shading. The first condition implies a fractal structure of dimension approaching 2. We show that the third condition is incompatible with both the first and the second. However, a QF tree with a slightly modified scaling in the vertical can, in the limit, satisfy the first and the last conditions perfectly, and also the second allowing for discontinuities of in-plane current densities. We then verify our theoretical analysis with a series of systematic and detailed experiments and simulations.

## Results

### Properties of QF hierarchical structures

To understand why fractal-like electrodes prove superior to other networks, consider a hypothetical ideal window electrode for PV applications. Since the efficiency of collecting current can always be increased at the cost of shading, assume fixed shading, defined as a fraction of the area of the light-harvesting surface. For such a constant shading, the theoretical ideal electrode would have the following three properties: (i) optimal coverage of the surface; (ii) uniform current density; and (iii) minimum in-plane resistance overall. Optimal coverage, whereby current is effectively collected from the entire surface, requires a progressively finer network of branches at smaller and smaller scales. If the overall network is to be near homogenous, a self-similar, surface-covering structure is required. Thus, condition (i) implies fine structure and self-similarity (fractal network) in the plane. Furthermore, near-perfect coverage of the surface implies a fractal of dimension *D* approaching 2. The simplest network is one with the branching number *N*=2, which is a pattern where at each order a given parent branch splits into two daughter sub-branches, a fractal tree^21^,^22^,^23^,^24^.

Consider first, for simplicity, an electrode in the form of a fractal tree with the square cross-section (the actual geometry of the cross-section does not matter for any of the results below, although it may affect the quality of the electrical contact). At a junction of order *n* of such a tree, a branch of width *r*_*n*_ and length *l*_*n*_ splits into two branches of widths and lengths *r*_*n*+1_ and *l*_*n*+1_, respectively (it is sufficient here to consider trees with identical daughter branches at each junction). Continuity of current through the junction, assuming fixed current density *J*_*o*_, condition (ii), gives


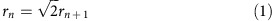


Consequently, the linear dimensions of the branches scale with the factor 1/*s* where 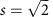, and the widths at each junction satisfy 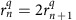 with *q***=**2. Uniformity of current density along each branch of the junction is now assured. Furthermore, since the area of contact between a given branch and the substrate is equal to the area of contact of its two daughter branches, 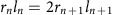, continuity of current guarantees also the uniformity of current density *J*_s_ from the substrate into the electrode. Indeed, 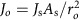, where *A*_s_ is the total shading area. The fractal dimension corresponding to this scaling is


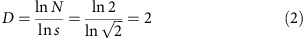


Evidently, conditions (i) and (ii) favour an electrode, which is a QF (truncated) tree of the fractal dimension approaching 2, in our hypothetical, idealized scenario.

We now turn to condition (iii). It has been shown rigorously that an electrode in the form of fractal tree has optimal properties for electrochemical applications, including minimal resistance^23^. Here we derive the exponent *q*, which minimizes electrical resistance with the in-plane cross-sectional area (shading) kept constant. To do this, we consider resistances of the branches meeting at a junction and employ the Lagrange multiplier method. The resistance of the parent branch at order *n* is given by 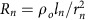, where *ρ*_*o*_ is the resistivity of the electrode material. Assuming the ratio *l*_*n*_/*r*_*n*_=*b* is a constant, 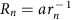, where *a*=*ρ*_*o*_*b* is a constant. It follows that the resistance of the segment of the network consisting of the parent and two daughter branches (at order *n*+1), and the area of its cross-section, can be written as 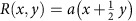 and *A*(*x, y*)=*b*(*x*^−2^+2*y*^−2^), respectively, with 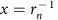, 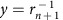. We therefore define the functional


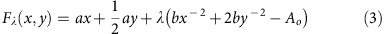


and seek its minimum. Setting the derivative with respect to the Lagrange multiplier *λ* to zero assures that the shading area is kept constant (and equal to *A*_*o*_). Setting the derivatives with respect to *x* and *y* to zero and eliminating *λ* from the resulting equations gives *x*^−3^=4*y*^−3^ or


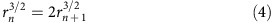


It follows that *q*=*D*=1.5 minimizes the net resistance, whereas *D*→2 is required for the uniform current density and optimal surface coverage. The two requirements cannot be satisfied simultaneously and thus a window electrode, of square or circular cross-section, which satisfies all three conditions formulated above, is impossible.

However, a branch cross-section does not need to be defined by a single parameter, and in practice it is quite unlikely to be. To take the simplest example, we assume that each branch has a rectangular cross-section, with width *r*_*n*_ and height *h*_*n*_. Furthermore, uniformity of current density is not physically necessary (as long as current is continuous as demanded by Kirchhoff's Law). By relaxing condition (ii), allowing discontinuities in current density across the junctions, and by controlling relative thickness of branches *h*_*n*_ at each order, one can design a network whose projection onto the cell's plane is a fractal of the desired fractal dimension. One way to do this is to set the height of the branch at *n*-th order to scale as


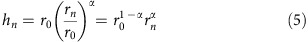


and require that the projection onto the plane of the cell be a (perfect) fractal of dimension *D*<2. Imposing condition (iii) for the scaling (5) now gives


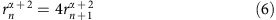


that is, *D*=(*α*+2)/2 or *α*=2(*D*−1). As expected, with *D***=**1.5, *α*=1 and (5) reduces to *h*_*n*_=*r*_*n*_ for square electrodes. Continuity of current requires now *J*_*n*_*r*_*n*_*h*_*n*_=2*J*_*n+*1_*r*_*n+*1_*h*_*n+*1_, which, on using (5) and (6) yields


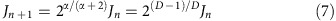


For the hypothetical ideal network, *D*=*α*=2, implying perfect coverage of the surface, height scaling given in our model by 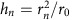, and current density increasing at each junction according to 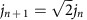. Now conditions (i) and (iii) are satisfied exactly, whereas condition (ii) assures continuity of current. A finite, real version of such a network would be optimal (save for the uniformity of current density), though of course highly cumbersome to manufacture.

It follows that our electrode based on the venation of a Magnolia alba leaf^18^, with the measured value *D*=1.4±0.2≈1.5, is close to the best achievable under the constraint of equal width and height of branches, with small discontinuity in current density at each junction (of about 25% using our model). The only deficiency of this network is the less than maximal surface coverage.

### QF network of leaf venation

**Figure 1:**
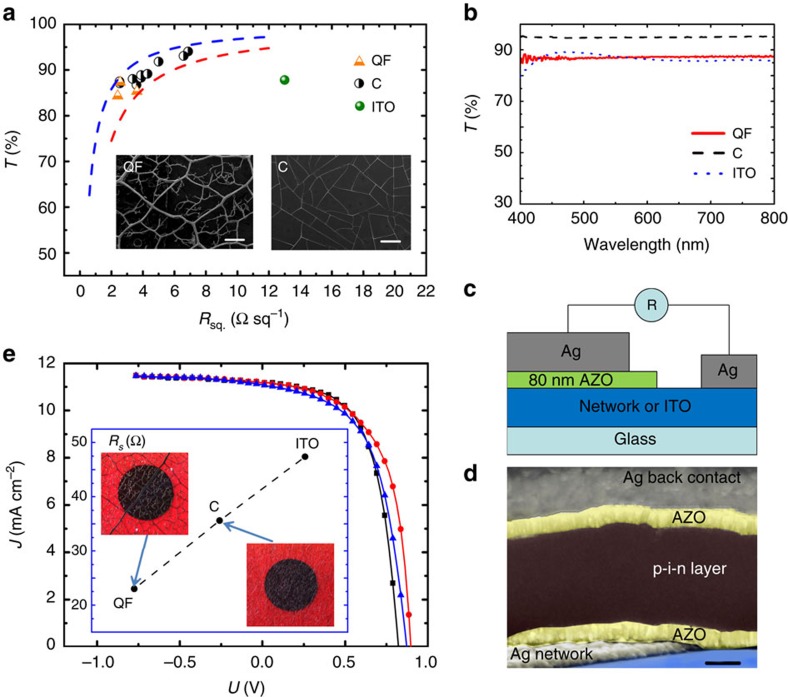
Optoelectronic performance and morphology of the networks and ITO films. (**a**) *T* versus *R*_sq_; *T* is measured at 550 nm vacuum wavelength. The expected analytical dependencies (equation (8)) for two figures of merit *F*=1,200 and 700 are shown as the blue and red dashed lines, respectively. The left inset shows the SEM image of the QF network based on the leaf venation (scale bar, 500 μm), and the right inset is the SEM image of the C network (scale bar, 100 μm). (**b**) *T* versus wavelength for QF, C and ITO films. (**c**) Schematic of the contact resistance measurement. (**d**) SEM image of the cross-section of the PV cell. The scale bar is 200 μm. (**e**) *I*–*V* characteristics of the PV cells under AM1.5 illumination: QF (red line with circles); C (black line with squares); and ITO (blue line with triangles). The inset shows a plot of the corresponding series resistance *R*_s_. Two optical images of the PV cells are also shown in this inset; the cell based on the QF network has a clearly visible leaf venation structure. The dark circle (0.196 cm^2^) in the middle of each cell is the Ag back reflector.

To demonstrate experimentally that a fractal-like network outperforms a non-hierarchical structure, we use the QF network developed earlier by members of our team and described in detail in ref. 18. It is based on the metalized (by sputtering of silver) venation of a Magnolia alba leaf. The left inset in Fig. 1a shows an scanning electron microscopy (SEM) image of this QF network. For comparison, we chose a non-hierarchical structure of the self-assembled ‘crack' (C) network^25^, shown in right inset in Fig. 1a, as well as the conventional ITO film. The C network is made by coating a substrate with a thin, sacrificial film, which micro-cracks under certain conditions, and acts as a mask during sputtering of a metal (cracking template). The lift-off of the sacrificial film produces the desired electrode network. In this work we modified our original process^25^ by using a much cheaper and environmentally friendly new material, egg white, as the sacrificial layer. Normally, we can roughly control the fabrication process and obtain networks with different wire width, inter-wire distance and the thickness of metal layer (Supplementary Table 1), also could fabricate large-area uniform samples (Supplementary Fig. 1). The experimental data points (transmittance *T* versus the in-plane sheet resistance *R*_sq_) for our QF and C networks, and ITO film, are shown in the main part of Fig. 1a. Dashed lines in this plot represent the well-known analytical formula.^25^,^26^,^27^


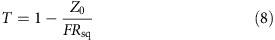


Where *Z*_0_ is the vacuum impedance, for two values of *F***=**1,200 (blue line) and 700 (red line). *F* is the figure of merit, often used to quantify transparent conductor performance. This formula can be easily derived by modelling a network as a uniform thin film, with a large, imaginary dielectric function, and by applying the standard Fresnel optics^27^. The large values of *F* for both QF and C networks (with data points ‘locked-up' between the dashed lines) indicate their very high quality as transparent conductors operating in the in-plane electron transport mode, out-performing the conventional ITO-based electrode. Figure 1b shows plots of *T* versus wavelength, indicating that the QF and C networks have a large and essentially frequency-independent *T*. Note that C networks slightly outperform QF networks in the figure of merit test, since these are optimized for the vertical, not the in-plane transport.

To test the optoelectronic performance of the films in the vertical electron transport mode (into or from a substrate), we have performed two additional experiments. In the first, we measure the contact resistance *R*_cont_ of a film (for example, a network) to the 80 nm thick, flat Al-doped zinc oxide electrode, as shown schematically in Fig. 1c. All films have been selected to have essentially identical *T*. As expected from theoretical analysis, the QF network in the vertical transport mode strongly outperforms both the C-network and ITO: *R*_cont_**=**2.6Ω (QF); 14.8Ω (C); and 28.4 Ω (ITO). In the second experiment, we developed a series of PV cells with window electrodes based on those films. Again, all electrodes have been selected to have essentially identical *T*. Since depositing the window electrode (QF, C or ITO) on a glass substrate was the initial stage of the cell development, it allowed us to select the electrodes based on *T*. Figure 1d shows an SEM image of the cross-section of the cell (the cell processing and *I*–*V* characteristic details in the Supplementary Methods). The identical processing has been used for all cells, and the completed cells have been finally selected to have identical short circuit current density and the shunt resistance, which is expected if the junctions are identical and all the window electrodes have the same *T*. The measured *I*–*V* characteristics of the selected PV cells under AM1.5 illumination are shown in Fig. 1e. The inset shows that as expected, the QF window electrode provides the smallest series resistance *R*_s_=23 Ω, 35% smaller than the random C electrode with *R*_s_=35.6 Ω, even though the same amount of metal was used (the same *T* implies the same metal coverage *ν*). Both QF and C networks have much smaller series resistances than the uniform thick film of ITO, with *R*_s_=47.4 Ω. The energy conversion efficiencies of 5.91%, 5.46% and 5.37% for QF, C and ITO cells, respectively, reflect this trend also. Note that the relatively low overall efficiency of the cells is a result of missing light-trapping schemes, omitted intentionally to emphasize the electrode performance. The additional insets in Fig. 1e show photographs of the PV cells, with clearly visible leaf venation structure for the QF network cell.

### Model networks

**Figure 2:**
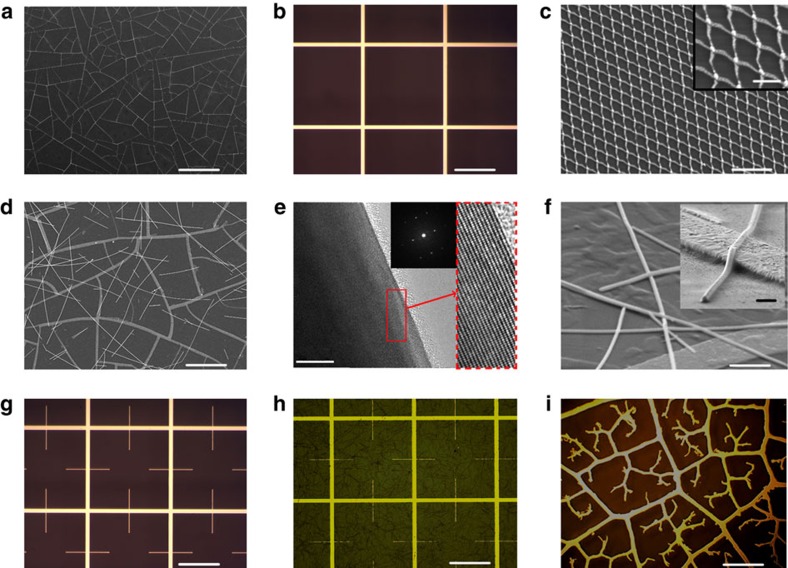
Morphology of the model networks. Single-order networks: (**a**) C network (R1; SEM image; scale bar, 200 μm); (**b**) large-scale simple grid network (P1; optical image; scale bar, 200 μm); and (**c**) ultrafine scale grid network (UP1; SEM image; scale bar, 5 μm). Two-order random networks: (**d**) C-based network (R2; SEM image; scale bar, 30 μm). (**e**) High (atomic)-resolution transmission electron microscopy images of a single NW. Inset shows an X-ray diffraction pattern. Scale bar, 10 nm. (**f**) High-resolution SEM image of the R2 network at the tilt angle of ∼60°. Scale bar, 2 μm. Inset: details of the ribbon–NW contact (scale bar, 400 nm). (**g**) Two-order periodic network (P2; optical image; scale bar, 200 μm). (**h**) Three-order periodic network (M3) obtained by adding NW to the P2 network. Scale bar, 200 μm. (**i**) Optical image of the quasi fractal QF network. Scale bar, 500 μm.

Our theoretical analysis, based on the network topology, shows that the geometrical details of the network play a secondary role. This implies that the increasing number of hierarchical orders, not network symmetry (periodic or non-periodic), should be the main consideration for a hierarchical network optimization. To test this conclusion experimentally, we developed a number of networks with varying network geometries, and a number of hierarchical orders (the schematic of the fabrication process is shown in Supplementary Fig. 2). Morphology of these networks is shown in Fig. 2. The first network (Fig. 2a) is the C network^25^,^28^,^29^, already discussed above. This is a first-order random network (R1). Figure 2b presents an optical image of a large-scale simple grid network. This is a first-order periodic network (P1). Figure 2c is an SEM image of an ultra-small scale grid network (UP1), developed to study plasmonic refraction, discussed later. This is a first-order ultrafine periodic network. The second-order random network (R2) is made by depositing silver nanowires (NWs) (the detail of synthesis of Ag NWs in Supplementary Methods) on the R1 network. Its SEM image (Fig. 2d) reveals the two-order hierarchy: the C network (first order) and the silver NW (second order). NWs have an average length of ∼50 μm and diameter of ∼200 nm. Figure 2e presents a high (atomic)-resolution transmission electron microscopy study of a single NW, showing an excellent quality with clearly visible, defect-free atomic planes (in lower and higher magnification), as well as almost perfect X-ray diffraction pattern. Figure 2f is a high-resolution SEM image of the details of R2 network, further enhanced in the inset and an atomic force microscopy (AFM) image, showing height profile of this network (Supplementary Fig. 3). Figure 2g is an optical image of the second-order periodic network (P2), obtained by adding the narrow horizontal and vertical metallic lines (of 200 μm length) to the P1 network. P1 and P2 large-scale networks were made by photolithography (the details are shown in the Methods section and Supplementary Fig. 2).

**Figure 3:**
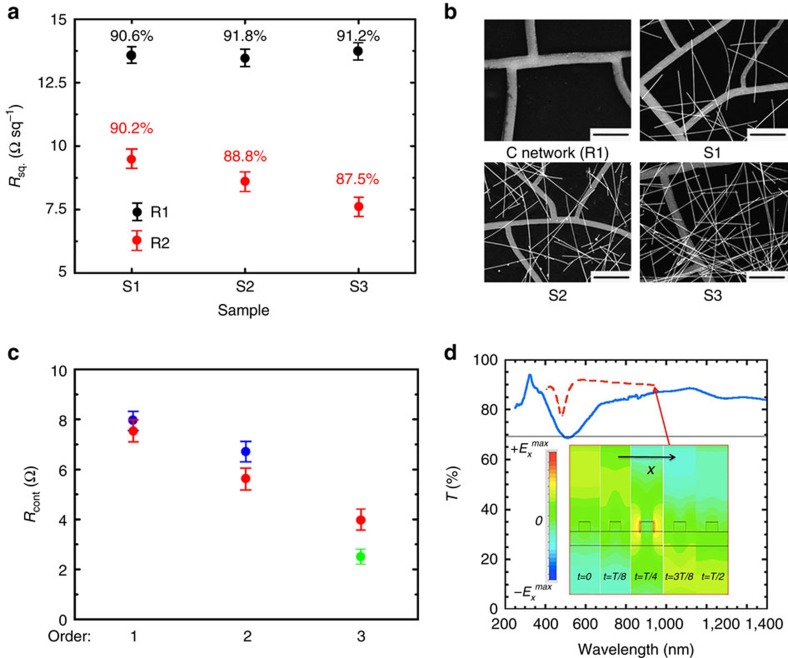
Optoelectronic properties and morphology of the hierarchical networks. (**a**) Measured *R*_sq_ and *T* of the C-based networks (black solid squares) and the corresponding R2 networks (red solid circles). The numbers at the data points are the corresponding *T* values in %. (**b**) SEM images of the C-based networks, with increasing NW density (C network: no NWs; S1: 0.125 mg ml^−1^; S2: 0.25 mg ml^−1^; and S3: 0.5 mg ml^−1^). The scale bars are all 10 μm. (**c**) Measured *R*_cont_ for hierarchical structures of various orders (each pair of symbols represents the same order). Blue circles represent random (R1 and R2) and red triangles periodic (P1 and P2) networks. The third-order networks are M3 (red circle) and QF (green square). (**d**) Transmittance of the ultrafine scale grid network (UP1) shown in Fig. 2c: experimental (solid blue line); simulated (dashed red line); and *T*_g_ (solid black). The inset shows maps of the x-component of the electric field near a single nanowire of the ultrafine network at various times, within the period *T*. The uncertainty in (**a**) and (**c**) comes from the non-uniformity of the film, and is calculated as a standard deviation.

To start with, a study was performed of the impact of the NW density on the in-plane network performance. The results are summarized in Fig. 3a, which shows (as red dots) sheet resistance *R*_sq_ of three, C-based R2 hierarchical networks called here S1, S2 and S3, each having increased number of deposited NW. SEM images of these networks, together with the corresponding NW-free C network, are shown in Fig. 3b. Clearly *R*_sq_ decreases strongly with increasing NW density, while the transmittance *T* (the numbers in % immediately above the data points) decreases much slower. This demonstrates the advantage of adding a new, lower order to a hierarchical network: while the transmittance is affected only marginally, because of a minimal shading increase due to the added fine structure, the resistance is lowered significantly due to optimization of the carrier transport via the network of ‘local roads'. Further confirmation of this advantage is via PV effect. We have made PV cells using the same procedure and structure as shown in Fig. 1d. As expected, the efficiency of the R2 cell is *E*_f_=6.83%, much higher than the efficiency of the best corresponding PV cell based on the C network (R1) (5.89%), and the ITO-based cell (5.21%). For more details see Supplementary Fig. 4. As before, the relatively low absolute values of the efficiencies reflect the absence of any light-trapping scheme omitted on purpose, to emphasize the electrode performance.

To test the importance of the network symmetry, we have made four additional networks, all with the same silver thickness of 100 nm on glass. The first is a C-based R1 network, with random structure similar to that in Fig. 2a, and the second is the C-based corresponding R2 network. The third network is a simple square network (P1) as shown in Fig. 2f, and the fourth, the two-order periodic hierarchical (P2) network based on this square network, as shown in Fig. 2g. To facilitate comparisons between these four networks, they were designed to have the same transmittance *T*∼90% (to within 2%), at the 550 nm vacuum wavelength. The contact resistance *R*_cont_ has been measured using the set-up of Figs 1c, and 3c shows *R*_cont_ versus network order, where the blue solid circles represent random and the red solid triangles periodic networks. As expected, the contact resistance of the periodic and the random networks of the same order, and having the same *T* is very similar. On the other hand, adding an order significantly reduces *R*_cont_. Note that while *R*_cont_ is very different for P1 and P2 networks, both networks must have exactly identical in-plane sheet resistances *R*_sq_, since the lower-order features of the P2 network form ‘dead-end' local ‘streets', which do not contribute to the in-plane transport. In addition to the networks discussed above, we also made a three-order hierarchical mixed network (M3) by adding NW to the P2 network. Optical image of this three-order mixed (periodic-random) network (M3) is shown in Fig. 2h. The corresponding contact resistance of this network is *R*_cont_∼3.7 Ω (red circle in Fig. 3c), less than other networks, except for the QF leaf venation network (∼2.6 Ω), shown in Fig. 2i. This confirms again the importance of increasing orders of hierarchical networks.

### Plasmonic effects

The networks utilizing NW have an additional benefit of the subwavelength scale of the NW in the visible frequency range. This provides conditions for plasmonic refraction effects^16^,^30^, which enhance the transparency above the classic super-wavelength geometric limit^17^ *T*_g_=1−*ν*, where *T*_g_ is the transmittance and *ν* is the surface fraction covered by metal. To demonstrate this, we calculated *ν* from the SEM images of the C-based R2 networks. While for the C network we confirmed that measured *T*≈*T*_g_, the statistically averaged *T*_g_ values for S1, S2 and S3 networks are 85.7%, 83.4% and 78.7%, respectively, all significantly smaller than the corresponding optically measured *T* values 90.2, 88.8 and 87.5%. To demonstrate that this enhancement is due to the plasmonic refraction, we use the ultrafine grid network (UP1), shown in Fig. 2c. The corresponding measured and simulated *T* versus wavelength for this network are shown in Fig. 3d. Even though these agree only qualitatively, both are substantially greater than the corresponding *T*_g_**=**69%, except for the immediate vicinity of *λ*=500 nm, where the localized Mie-like plasmon is excited^31^. To visualize the plasmonic refraction, we also simulated electromagnetic fields in the vicinity of a single NW at *λ***=**950 nm, that is, in the high-*T* region. The simulated snapshots of the x-component of the electric field (colour encoded) for various times *t* are shown in the inset in Fig. 3d. The wave-front evolves from the essentially plane wave at *t***=**0, to the complete partition of the wave-front at *t***=***T*/4, back to almost a complete recovery of the plane wave propagation past the NW for *t***=***T*/2. Clearly, only a very small back reflection occurs, as indicated by a tiny distortion of the wave-front at *t***=**0, with the electromagnetic wave avoiding, and propagating around the NW. Note that the field inside the wire is zero, at all times, confirming the classic nature of the wave–metal interaction; retarded surface plasmons follow dispersion very close to the light line, and in fact transition into it for vanishing frequency. Wave propagation around the minimum due to localized plasmons (at *λ***=**500 nm) is completely different, with standing waves forming, and the wave energy propagating through the NW.

## Discussion

The theoretical analysis presented here starts with three postulates for a hypothetically ideal window electrode: an optimal surface coverage; a uniform in-plane current density; and minimal overall resistance (at given shading). The first two of these conditions require a fractal structure of dimension approaching 2, which of course is an idealization. The third condition, implying *D***=**1.5, while manifestly incompatible with the first two, suggests that an electrode consisting of a truncated fractal structure of this dimension would be optimal except for less than perfect surface coverage. This explains why a network based on the veins of Magnolia alba leaf, with *D*∼1.4, proves to be exceptional—at least within the class of quasi fractal networks in three dimensions.

Indeed, in a series of detailed experiments, we showed that our ‘natural' electrode had a significantly higher PV efficiency and smaller contact resistance, than any uniform, non-hierarchical random network, as well as the conventional ITO layer, confirming our conclusions.

These results notwithstanding, the requirement of uniform current density is not really necessary, and in fact usually not satisfied in artificial networks. Furthermore, it is desirable only insofar as to assure uniform current density into and/or from the substrate, itself not critical. Mathematically, uniformity of current density means that the network must be a fractal tree in three dimensions. We therefore designed a network, which is not fractal as a whole, but whose projection onto the window's plane is fractal—and of any desired dimension *D*, up to and including 2. Continuity of current through every junction now determines the necessary jumps of current density. It follows that a truncated version of such a network, set to *D***=**2, would theoretically be optimal, though it may be impractical to manufacture.

Beyond this theoretical result, our analysis indicates that a hierarchal structure (with multiple branching orders), rather than detailed geometry, is critical for optimizing network electrodes. We demonstrated this experimentally in a series of systematic electro-optical and PV experiments, in five hierarchical networks: a one- and two-order random, one- and two-order periodic and a three-order mixed structures. Our experimental results are summarized in Fig. 3. Figure 3d, in particular, demonstrates this conclusion explicitly. At fixed transmittance, changing from random to periodic structures, while keeping the number of hierarchical orders fixed, lowers the contact resistance only slightly, but adding another hierarchical order, for both period and random structures, reduces it by 30–50%. All the while, the in-plane resistance remains essentially unchanged.

In addition to these structural effects, controlled by network hierarchy with topological elements (branching), the networks based on NWs achieve transparency exceeding the geometric limit. This is due to plasmonic refraction, which involves propagating surface plasmons transporting energy around NWs, effectively ‘squeezing' light into the spaces between NW, and thus minimizing reflection. We demonstrate this effect on our ultrafine grid network, whose transparency has been measured and simulated. To visualize the plasmonic refraction, we performed also simulations of the electromagnetic fields of waves propagating through the network at various times, and for varied wavelength. Our simulations show that an electromagnetic wave couples to the surface plasmon wave in the metal, which in turn transports its energy seamlessly around the NW. The net result is an increased transmission of the wave through the network.

Our low-cost, self-assembled networks are mechanically very stable and flexible, surviving numerous bending cycles (for an example see Supplementary Fig. 5 and Supplementary Methods). For comparison, the resistance of ITO film changes by two orders of magnitude already after a few cycles. Such desirable mechanical stability and flexibility, combined with excellent electro-optical parameters demonstrated here, make these networks very attractive. They are suitable, in particular, for solar cells, LED lighting and transparent flexible heaters (Supplementary Fig. 6). Our study opens a path towards a new generation of smart TC, through hierarchical multi-order networks with the lowest orders at nanoscopic scales, and possibly also through intrinsic TC functionalities, such as light trapping and/or nonlinear light processing. These smart TC could eventually lead to a new class of applications such as the energy recovery, temperature and haze controlling electro-chromic windows, and high-power light sources, to name a few.

Finally, we note that while our most efficient electrode to date is based on a natural biological network, the mathematical construct we developed suggests that a superior electrode can yet be envisioned, though it seems impractical at this stage.

## Methods

### Fabrication of random networks R1 and R2

A pure egg white was diluted with deionized water to the concentration of about 0.6 g ml^–1^. Spin coating (Spin coater, Laurell, USA) was used to deposit as-prepared egg white film on substrates. These were dried in air (temperature range 25–70 °C). After several minutes, the self-induced cracking process occurs. A set of samples, with averaged crack width of 0.5–5 μm and averaged size of the gel island of 20–500 μm, were obtained by varying the spin-coating speed (200–800 r.p.m.), coating time (10–50 s), concentration of sol-gel and the drying temperature. The cracking template morphology can be controlled with these fabrication parameters (details in Supplementary Table 1). The sputtering system (AJA International, ATC Orion 8, USA) was used to deposit Ag films, with thickness controlled in the range of ∼100 nm. Rinsing with deionized water for 1–2 min was used to remove the sacrificial layer of the egg white film. This way we made all the R1 C-based networks. The R2 networks were manufactured by coating R1 networks with different concentration of Ag NWs. The schematic of fabrication processes (R1 and R2) is shown in Supplementary Fig. 2a).

### Fabrication of hierarchical networks

Spin coating (Spin coater, Laurell, USA) was used to deposit as-prepared U-8 photoresist film on substrates. Next, the film was pre-baked in 90 °C, and then exposed to ultraviolet light for about 10 s through two different masks: one for the P1 and the other for the P2 network (details in Supplementary Fig. 2b,c). Finally, the sample was developed in the developing liquid for about 45 s. The sputtering system (AJA International, ATC Orion 8, USA) was used to deposit Ag films. The thickness of Ag film was about 100 nm. Rinsing with anhydrous alcohol for 1–2 min was used to remove the sacrificial layer of the photoresist film. The third-order M3 network was obtained by coating the P2 network with Ag NWs (details in the Supplementary Fig. 2d). UP1 structure was fabricated by using a modified nanosphere lithography, as described in detail in ref. 31. This technique, which produces an array of parallel lines was used twice, in combination with the sample rotation, to yield the final structure.

### Morphology

Morphologies of samples were characterized in a SEM system (JEOL JCM-5700, Tokyo, Japan), and by using an optical microscope (MA 2002, Chongqing Optical & Electrical Instrument Co.). High (atomic)-resolution transmission electron microscopy images were obtained using the transmission electron microscope (JEOL JEM-2100HR, Japan) operating at an acceleration voltage of 200 kV. AFM image and height profile was obtained with the AFM (Cypher, Asylum Research, USA) in a.c. mode.

### Transmittance measurements

Optical transmittance was measured in an integrating sphere system (Ocean Optics, USA). Transmittance measurements have been normalized to the absolute transmittance through the substrate Polybutylene terephthalate (PET) or glass.

### Electrical conductivity measurements

The sheet resistance *R*_sq_ measurements were obtained by using the van der Pauw method, as described in ref. 25. The contact resistance *R*_cont_ measurements were made by coating a half of a network sample with 80 nm of the Al-doped zinc oxide film, and then applying a fixed structure of contacts, as schematically shown in Fig. 1c.

### Simulations

The simulations of the transmittance and the field maps for the network UP1 used the finite difference time domain and finite difference frequency domain code CST (https://www.cst.com/products/CSTMWS).

### Data availability

The authors declare that all data supporting the findings of this study are available within the article and its Supplementary Information files.

## Additional information

**How to cite this article:** Han, B. *et al.* Optimization of hierarchical structure and nanoscale-enabled plasmonic refraction for window electrodes in photovoltaics. *Nat. Commun.* 7:12825 doi: 10.1038/ncomms12825 (2016).

## Supplementary Material

Supplementary Information Supplementary Figures 1-6, Supplementary Table 1 and Supplementary Methods.
