# Supplementary material for: Optimization of hierarchical structure and nanoscale-enabled plasmonic refraction for window electrodes in photovoltaics
Source: Nat Commun. 2016 Sep 26;7:12825. doi: 10.1038/ncomms12825 (PMC5052667; doi:10.1038/ncomms12825)
Supplement: Supplementary Information — Supplementary Figures 1-6, Supplementary Table 1 and Supplementary Methods. [file ncomms12825-s1.pdf]

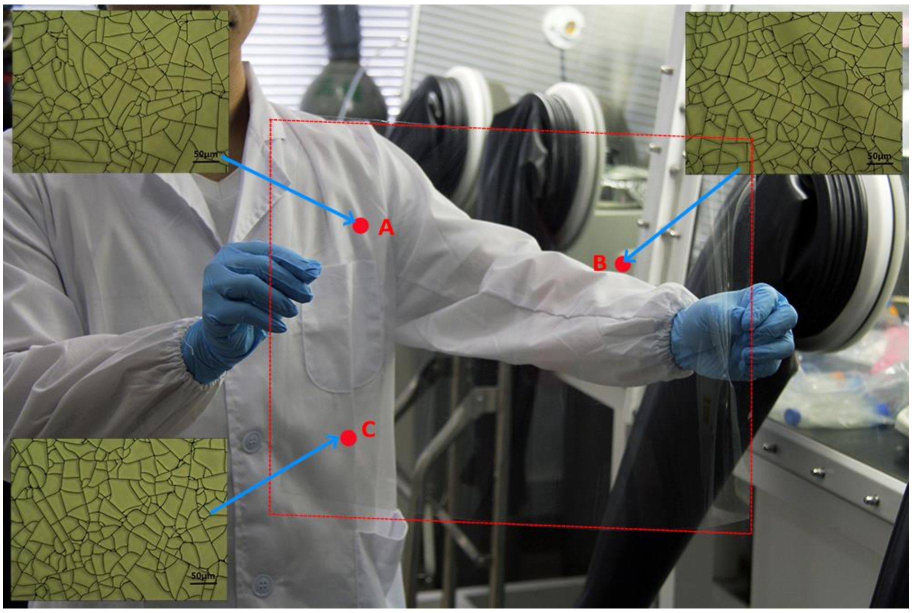


**Supplementary Figure 1** Photograph of the C network (R1) film on the PET substrate (0.6 m × 0.6 m).

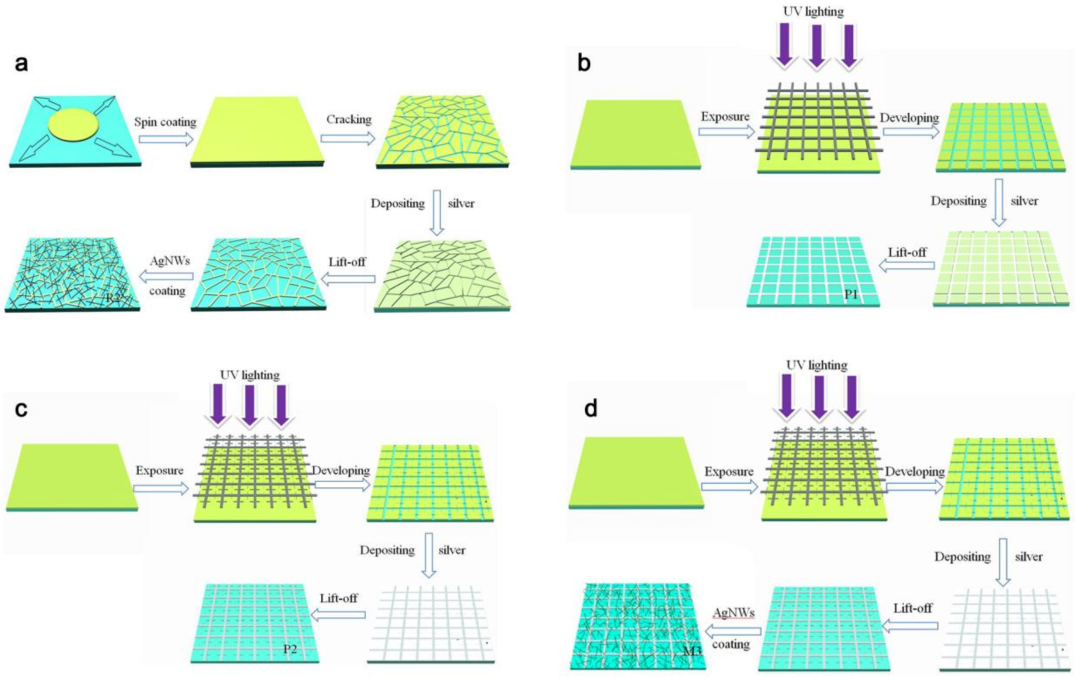


**Supplementary Figure 2** Schematic of fabrication hierarchical model networks a) C network (R1 and R2), b) P1, c) P2, and d) M3.

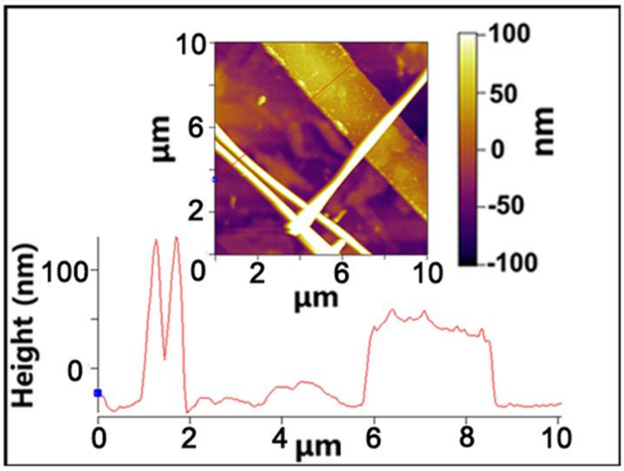


**Supplementary Figure 3** AFM image and height profile of the R2 network.

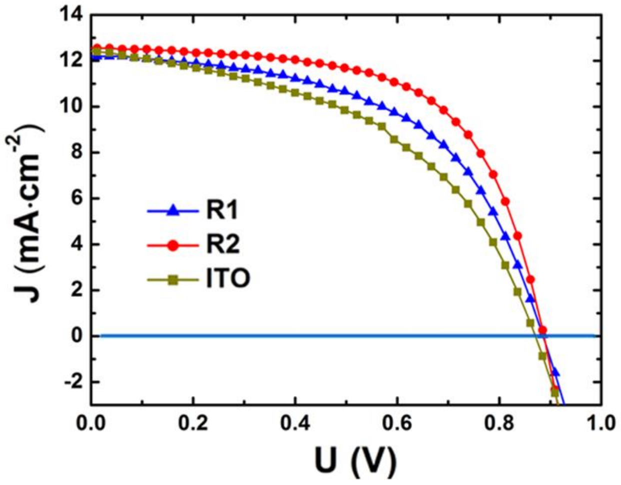


**Supplementary Figure 4** I-V characteristics of the PV cells with the R1 (blue line with solid triangles), R2 (red line with solid circles) and ITO (green line with solid squares), under one sun (AM1.5) illumination conditions. The efficiency of the R2 cell (based on the S1 network) is $E_f$ = 6.83%, much higher than the efficiency of the best corresponding PV cell based on the C network (R1) (5.89%), and the ITO based cell (5.21%).

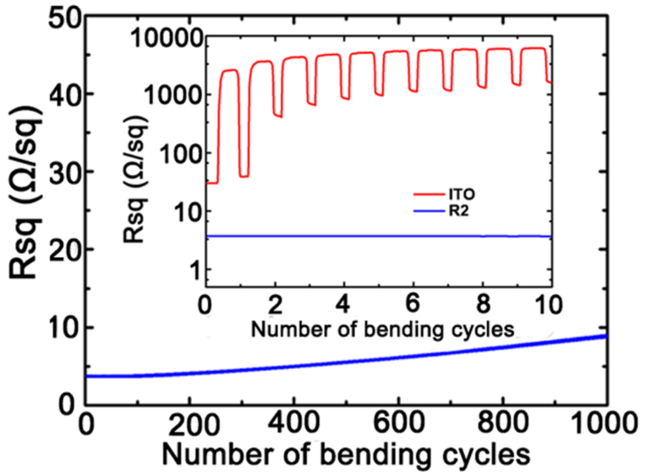


**Supplementary Figure 5** $R_{sq}$ versus number of bending cycles for the R2 network (blue lines), and the ITO film (red line, inset), showing no change up to 100 bending cycles, and only minimal change thereafter (doubling after 1000 cycles). For comparison, the resistance of ITO film (shown in the inset) has been increased by two orders of magnitude after a few cycles.

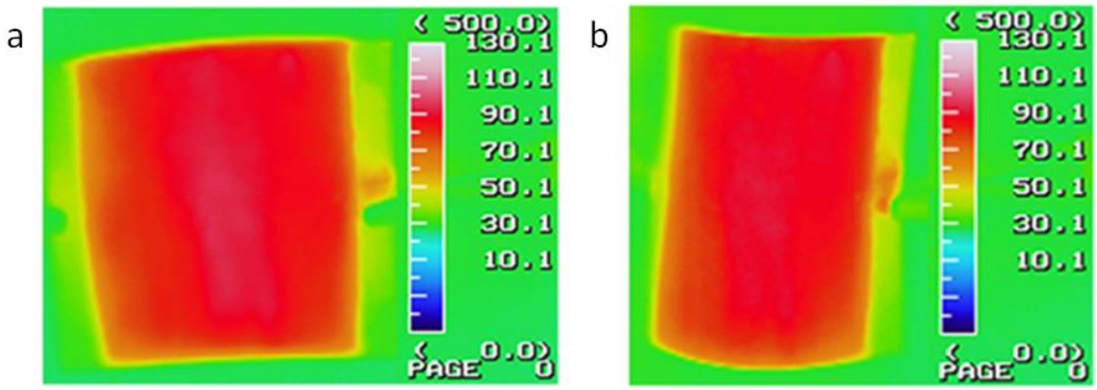


**Supplementary Figure 6** IR image of the R2 film (heater mode): (a) before, and (b) after bending, confirming the minimal affect of mechanical bending on the electrical conductivity.

**Supplementary Table 1** The processing parameters, resulting network structures and their physical properties for the C network: cracking wire width (CWW), mean gel-isle size (GI), averaged silver wire width (SWW), inter-wire distance (IWD), silver film thickness, and area coverage of metal film, and their physical properties (sheet resistance and light transmittance). However, we should mention that all the experiments are done by solution-process methods, the morphologies of those random structures are estimated via these microscopic images.

| Samples | Concentration of egg white (g/ml) | Speed (rpm) | Time (s) | CWW/ SWW (μm) | GI/IW D(μm) | Area coverage of metal film (%) | Thickness of metal film (nm) | $R_s$ (Ω/sq) | $T$ (%) |
|---|---|---|---|---|---|---|---|---|---|
| 1 | 0.6 | 800 | 50 | ~0.5 | ~470 | ~0.21 | 80 | 28 | 99.4 |
| 2 | 0.6 | 800 | 20 | ~0.5 | ~180 | ~0.56 | 80 | 20 | 99.0 |
| 3 | 0.6 | 400 | 20 | ~2 | ~62 | ~3.25 | 80 | 7.7 | 95.8 |
| 4 | 0.6 | 800 | 10 | ~1 | ~133 | ~1.50 | 80 | 17.3 | 97.5 |
| 5 | 0.6 | 400 | 10 | ~2 | ~40 | ~5.1 | 80 | 6.2 | 93.0 |
| 6 | 0.6 | 200 | 10 | ~5 | ~50 | ~10.25 | 80 | 3.9 | 88.0 |
| 7 | 0.6 | 200 | 10 | ~5 | ~50 | ~10.25 | 150 | 3.7 | 88.0 |
| 8 | 0.6 | 200 | 10 | ~5 | ~50 | ~10.25 | 40 | 9.0 | 88.6 |
| 9 | 0.6 | 200 | 10 | ~5 | ~50 | ~10.25 | 10 | 39.6 | 90.2 |

**Supplementary Methods**

**Synthesis of Ag nanowires.** 50 mL of ethylene glycol (EG, Da Mao, Tianjin, China) in a glass flask was preheated to 160 °C, keeping ~30 min under continuous magnetic stirring. 0.36 g of PVP (Aladdin, 9003-39-8) was dissolved in 25 mL EG. Also 0.4 g $AgNO_3$ (Xi Long, Guangdong, China, 7761-88-8) was dissolved in 25 mL EG. Both processes were done under magnetic stirring at room temperature, and subsequently, the two solutions were mixed together. Before adding the mixture ($PVP+AgNO_3$) into the preheated EG in flask, 140 μL of 6 mM $FeCl_3$ (Aladdin, 7705-08-0) solution (in EG) was added to the mixture and stirred for two minutes, followed by heating to 150 °C for 20 min. Then the reaction temperature was retained at 135 °C for 3 h until the reaction was complete (without stirring).

**Fabrication of the a-Si PV cell.** The clean substrates (with networks or ITO)were installed into vacuum chamber ($5\times10^{-8}$ Torr) of the PECVD system (Solasta, Boston, USA). These substrates were exposed to gas mixture consisting of $B_2H_6/SiH_4/CH_4/H_2$, resulting in the ~ 10 nm thick p-doped *a*-Si layer. Subsequently, the mixture of $SiH_4/H_2$was used to make ~ 450 nm thick i-layer of *a*-Si, and the mixture of $PH_3/SiH_4/H_2$to produce ~ 50 nm thick n-layer of *a*-Si. Finally, the 80 nm thick buffer layer of AZO, followed by 300 nm continuous Ag film (as the back contact) was deposited by sputtering (AJA International. ATC Orion 8, USA).

**Bendability and Infrared (IR) image measurement.** A simple setup of the two-probe electrical contact (two fine silver paste lines (2 cm in length, 2 cm in separation) are brushed on samples), combined with a lab-made automatic bending machine was used to test the resistance change with the bending cycles of the networks, with an automatic control and data recorded with a Keithley 2400

Sourcemeter. Infrared image is recorded with the infrared thermal imager (NEC San-ei Instruments, Ltd, Japan).

**IV characteristics.** Solar cells were characterized under simulated AM1.5 sunlight at 101 mW $cm^{-2}$ irradiance generated by simulator (Newport 92193H-1000, USA).
